# Social Media Applications: A Potential Avenue for Broadcasting Suicide Attempts and Self-Injurious Behavior

**DOI:** 10.7759/cureus.10759

**Published:** 2020-10-01

**Authors:** Garrett Rossi, Roshi DeSilva

**Affiliations:** 1 Psychiatry, Cooper University Hospital, Camden, USA; 2 Psychiatry, Cooper Medical School of Rowan University, Camden, USA

**Keywords:** social media, effects of social media, social psychiatry, psychiatry, suicide prevention, suicide risk, bullying and suicide, impact of suicide

## Abstract

Social media is a mainstay in American culture, but it’s impact on mental health continues to evolve. Social media has revolutionized the way people share information and communicate. It is particularly popular among adolescents and young adults ages 18-24. There has been increasing concern that the internet and social media may be influencing suicidal behavior and self-harm in this population. Publications focusing on the impact of social media use on suicidal/self-injurious behavior are beginning to emerge in the literature. This clinical vignette chronicles the events of a suicide attempt documented on the social media application Snapchat. It facilitates a discussion about the role social media played in influencing this behavior.

## Introduction

Social media has emerged as a novel avenue for people to discuss mental health issues. Social media platforms are able to reach a wide audience and can engage difficult to reach populations. They provide an anonymous, easily accessible location to share experiences. Social media platforms have a well-documented history of being used to express suicidal thoughts and feelings. Adolescents and young adults ages 18-24 who self-harm use the internet more often than similar controls [1]. Adolescents and young adults who self-harm tend to use internet forums as a primary means of communicating distress. There is an association between suicide-related searches and the incidence of suicide [2]. Postings on the social media application Twitter that contained certain keywords were linked to increased incidence of suicide [3]. Postings on social media can influence behavior by normalizing, triggering, creating competition, and causing a contagion effect [4]. Normalizing makes self-harm seem like a regular and effective coping strategy for distress. Triggering occurs when a person considering suicide or self-harm views material of someone else engaged in these behaviors then acts on their thoughts. Competition is created by tracking the metrics offered on social media sites such as “likes” and the total number of views on a post. The contagion effect can occur when these social media posts reach a wide audience and spread precipitously to vulnerable populations. Comments made by other users have the ability to negatively influence the person making the post [5]. Comments that are cynical or encourage suicide or self-harm may result in perpetuation of these behaviors or death, as demonstrated in the case below.

## Case presentation

The patient presented to the emergency department (ED) status post intentional overdose of oxycodone/acetaminophen, alcohol, and diazepam. Upon arrival to the ED, he required physical restraints and medication for severe agitation. The patient's vital signs were stable, physical exam and labs were largely unremarkable. However, his blood alcohol level was 111, and a urine drug screen was positive for benzodiazepines, opiates, and cannabis.

Once calm, he was evaluated by the emergency psychiatrist and stated, “I made a bad decision”. An altercation occurred early in the morning between the patient and his girlfriend, which resulted in the police coming to his residence. Prior to the police arriving, he reported ingesting several pills, marijuana, and alcohol. The police asked him to leave the apartment so his girlfriend could gather her possessions. When he arrived back at the apartment, all the furniture was destroyed. He then left the apartment and went to his mother's home, where he located her pain medication and proceeded to take all of it. He does not recall any of the events that occurred thereafter. All of these events occurred over the course of a single day.

The patient's brother was available to provide collateral information. He showed a series of social media postings made on the social media application Snapchat. The posts included the patient taking fifteen 10 mg diazepam tablets, eating marijuana, and consuming one pint of rum. The last video ended with the patient consuming ten tablets of oxycodone/acetaminophen stating, “There is no coming back from this”. There were several thousand views of these events with some commenters encouraging the behavior, while others recommended going to the hospital. Based on the patient's account, the views and comments on social media influenced his behavior. At this point, the treatment team became interested in the connection between social media use and suicide.

Upon a more detailed examination, the mental status examination revealed a well-developed and well-nourished person. The patient spoke eloquently, with a normal rate, rhythm, and volume. He was calm and cooperative during the evaluation. His mood was “okay”, and his affect was restricted. He denied suicidal or homicidal ideation with intent or plan. He denied auditory or visual hallucinations. His thought process was linear. He was remorseful at the time of evaluation but lacked the insight to understand the severity of his actions. He assured the treatment team of his safety, but the impulsive nature and influence of social media on his actions demonstrated poor judgment.

The differential diagnosis included substance-induced mood disorder, major depressive disorder, bipolar disorder, intermittent explosive disorder, alcohol use disorder, cannabis use disorder, and opioid use disorder. Given the severity of the suicide attempt and the patient's lack of insight into the dangerousness of his behavior, an involuntary commitment was required. 

## Discussion

Research exploring internet use, social media, in particular, as an outlet for broadcasting footage or images of self-harm, is a growing area of interest. As the influence of the internet on self-harm and suicide becomes more apparent, there is now increasing concern on the part of parents, teachers, and government officials. 

The internet has provided an environment that makes us feel more connected while interacting with others. Social networking sites like Facebook, Snapchat, and Instagram allow individuals to communicate with others who share similar views. These platforms can provide therapeutic support to people struggling with self-injurious behavior (SIB) [1]. However, some of the individuals interacting on these sites are accepting of SIB and may be minimizing the potential risk of these behaviors. 

Daine et al.performed a systematic review of the influence of the internet on self-harm and suicide among young adults. They concluded that young people who self-harm are also frequent users of the internet [1]. They found both positive and negative influences of the internet on suicide and self-harm. The positive benefits include access to support groups and coping strategies. Negative influences include normalizing self-harm and discouraging the use of professional help. Marchant et al. performed a systematic review of the relationship between internet use, self-harm, and suicidal behavior in young people. Forty-six independent studies of varying quality were included, and the results were divided based on whether or not the influence was positive, negative, or mixed. They found that there is significant potential for harm from online behavior, including normalization, triggering, competition, and contagion, but as in other similar reviews, there are potential benefits as well. These benefits include crisis support, reduction of social isolation, delivery of therapy, and outreach [6]. Regardless of the potential dangers or benefits, young people are using social media to communicate distress. It would be wise for any clinician working with young people to ask about the use of the internet and social media. These conversations can identify potentially dangerous behavior and open up opportunities to provide early intervention. The connections users make on social media sites may be valuable to explore for therapeutic benefit. 

Hagihara et al. looked at the association between suicide-related searches on the internet and the incidence of suicide. They concluded that internet searches for specific suicide-related terms are correlated with the incidence of suicide among 20-30-year-old individuals in Japan [2]. Routinely asking about searching for or visiting these websites might lead to a reduction in the incidence of suicide among this population. Despite a significant increase in social media use, especially among young adults, there is little research on how people use these methods to communicate their behaviors [5]. 

Sueki looked at the association of suicide-related Twitter use with suicidal behavior [3]. Using logistic regression, the study showed that tweeting “want to die” and “want to commit suicide” was significantly related to suicidal ideation and suicidal behavior. Having a Twitter account and tweeting daily were not associated with suicidal behavior. This study provides evidence that Twitter logs may provide a way to identify internet users who are suicidal. This may lead to earlier identification and possible crisis intervention. 

The use of social media sites, such as YouTube, to post videos and receive comments or responses may lead to the maintenance of suicidal/self-injurious behavior. The people posting these videos may be doing it for several reasons, including connecting with people going through similar situations and searching for resources that may aid in recovery. A review of comments posted in response to non-suicidal self-injury (NSSI) YouTube videos conducted by Lewis et al. helps to determine the impact of these comments and the potential for recovery-oriented social support. They looked at comments posted in response to the 100 most-viewed NSSI videos posted on YouTube and found that the most frequent comments were self-disclosure, followed by feedback for the video uploader, or encouragement for the video uploader. A majority of the self-disclosure comments did not mention recovery at all, and in fact, revealed they were still self-injuring [7]. They found that their results suggested that viewer responses to the videos may result in maintenance of the behavior and that they rarely mention recovery. 

Ma et al. explored the concept of “dying online” and examined live broadcasts of suicides among the Chinese ages 18-25. They identified five stages of suicide incidents blogged online, which included: signaling, initial reactions, live blogcast of suicide attempts, crisis responses, and final outcomes [8]. Furthermore, they identified the role of the audience in these blogged attempts as consoling, providing information for timely rescues, reporting to the police and rescue, cynical and indifferent attitudes, and even “likes” and incitement. This analysis of multiple case studies was able to better identify the stages at which these broadcasted incidents occur and how the interaction with the audience typically results. Although participants in these blogged attempts typically appeared to be positive, there was an audience recognized that would encourage suicidal behavior. Further study would potentially allow for the development of intervention integrated into social media platforms. 

Robinson et al. identified 30 unique studies via systematic review, which focused on the ways that social media platforms could be used in suicide prevention. Four studies described the development of social media sites for suicide prevention, six studies described the potential of social media in its ability to reach or identify people at risk of suicide, fifteen looked at how people were using social media for suicide prevention-related purposes, and five examined experiences of those people who had used social media sites for suicide prevention [9]. Although there are multiple positive implications of suicide prevention in the context of social media, there is also a discussion for the potential hazards. For example, there are obvious limitations associated with social media accounts in terms of ethics and confidentiality. Additionally, there is concern that individuals will utilize social media platforms as opposed to professional support offline. There is also the potential for normalizing suicide-related behavior [10]. There were no studies reported on the development or findings of intervention for suicide prevention using social media. However, it seems that this would be a popular topic of exploration in the years to come. 

## Conclusions

There is significant potential for harm among young adults related to the use of social media as an outlet for expressing distress or suicidal ideation. It provides a means of normalizing self-injury/suicidal behavior. It may also trigger young people who are at risk for suicide, or self-injury, to perform life-threatening acts. It can create an atmosphere of competition where young people attempt to one-up each other resulting in serious injury, or even death. These behaviors can spread rapidly to a wide audience. It is well established that young people are using social media to communicate distress, and suicidal/self-injurious thoughts and behaviors. The focus of the clinician should be to inquire about the problematic use of social media in young adults. This can be accomplished in crisis centers, in the inpatient or outpatient settings. 
